# **Enhancing tomato plant immune responses to Fusarium wilt disease by red seaweed Jania **sp.

**DOI:** 10.1038/s41598-024-67233-0

**Published:** 2024-08-05

**Authors:** Amer M. Abdelaziz, Ahmed A. Elrefaey, Mohamed H. Sharaf, Rahmah N. Al-Qthanin, Mohamed S. Attia

**Affiliations:** 1 Botany and Microbiology Department, Faculty of Science, Al-Azhar University, Cairo, 11884 Egypt; 2 Biology Department, College of Sciences, King Khalid University, Abha, Saudi Arabia; 3 Prince Sultan Bin-Abdul-Aziz Center for Environmental Researches and Natural Resources Sustainability, King Khalid University, P.O. Box 960, 61421 Abha, Saudi Arabia

**Keywords:** *Jania*, Antioxidant, Defense, Isozyme, Tomato, *Fusarium*, Biotechnology, Microbiology, Plant sciences

## Abstract

The novelty of this study lies in demonstrating a new approach to control wilt diseases using *Jania* ethyl acetate extract. In the current investigation, the potential impacts of *Jania* sp. ethyl acetate extract (JE) on Tomato *Fusarium oxysporum* wilt (FOW) have been studied. The in vitro antifungal potential of JE against *F. oxysporum* (FO) was examined. GC–MS investigation of the JE revealed that, the compounds possessing fungicidal action were Phenol,2-methoxy-4-(2-propenyl)-,acetate, Eugenol, Caryophyllene oxide, Isoespintanol, Cadinene, Caryophylla-4(12),8(13)-dien-5à-ol and Copaen. *Jania* sp. ethyl acetate extract exhibited strong antifungal potential against FO, achieving a 20 mmzone of inhibition. In the experiment, two different methods were applied: soil irrigation (SI) and foliar application (FS) of JE. The results showed that both treatments reduced disease index present DIP by 20.83% and 33.33% respectively. The findings indicated that during FOW, proline, phenolics, and the antioxidant enzymes activity increased, while growth and photosynthetic pigments decreased. The morphological features, photosynthetic pigments, total phenol content, and antioxidant enzyme activity of infected plants improved when JE was applied through soil or foliar methods. It is interesting to note that the application of JE had a substantially less negative effect on the isozymes peroxidase and polyphenol oxidase in tomato plants, compared to FOW. These reactions differed depending on whether JE was applied foliarly or via the soil. Finally, the use of *Jania* sp. could be utilized commercially as an ecologically acceptable method to protect tomato plants against FOW.

## Introduction

Among the most daunting challenges for farmers is to feed the ever-increasing global population, which is expanding at a rate of about 1.05% per year, amid increasingly globalized climatic and natural disturbances. To overcome these obstacles, researchers estimate that by 2050, it might be important to increase the yield of essential food crops by about 87 present .

FOW adversely impacts crop yield, significantly decreasing both quantity and quality^1^. It must consider that, some fungal species can have a detrimental influence on crops and plants. Among them is FO, which causes critical plant disease in Egypt, reducing reproduction and negatively affecting economic benefits. Fungal infections indirectly contribute to the build-up of reactive oxygen species (ROS) such as singlet oxygen, superoxide radicals, and H_2_O_2_. Increased oxidative stress limits vital processes such as transpiration, water absorption and nutrient uptake dynamics as well as chlorophyll biosynthesis . Plants have developed adaptive responses to survive under fungal infection, which include development of morphological, physiological and metabolic adaptations^2^.

Several studies have evaluated the impact of eco-friendly materials as effective strategies to minimize damage effects by fungal diseases, aiming to increase crop production. Bio-stimulants improve plant growth and resistance to stress by enhancing nutrient quality and boosting soil nutrient uptake. Currently, scientists are focusing on biological control using plants, algae, bacteria, and fungi^4–6^. These microorganisms, metabolites are usually responsible for inhibiting fungal growth.

Marine macroalgae are aquatic plant-like organisms, categorized as green, brown, or red depending on their pigmentation. These algae are regarded as one of the most extensive and interesting sources of bioactive metabolites, comprising pigments, amino acids, polysaccharides, alkaloids, flavonoids, saponins, aromatic compounds, peptides and terpenes, These substances have biological functions such as bactericidal and fungicidal activities^7^. Recently studies have emphasized marine macroalgae as a biocontrol agent against fungi through various metabolites as pigments chlorophyll a, phycobilins, alkaloids, phenolics, terpenoids^8^. Red algae are distinguished by pigments chlorophyll a, phycobilins and carotenoids, mostly β-carotene, lutein and zeaxanthin. The red alga *Jania* was used for the biocontrol of plant pathogens^9,10^.

This study aimed to evaluate the role of JE treatment in enhancing the immune responses of tomatoes against FOW by modifying vegetative development and physiological features under two distinct conditions: foliar and root application.

## Materials and methods

### Algal material collection and identification

Hand picking was used to gather macroalga from the Red Sea in Hurghada, Egypt. The red alga was identified according to Norris^11^. The algal was cleaned by tab water to remove the epiphytes, salt minerals, and debris.

### Macroalgal extract preparation

To prepare an ethyl acetate extract of marine macroalga, marine, the macroalga was, dried in an oven at 35 ℃ and extraction process performed according to Somasekharan et al.^12^, The crude extract (10 mg/ml) was then stored at 4 ℃ as a stock solution.

### Fungicidal action of algal extract

The fungus *Fusarium oxysporum f.* sp. *lycopersici* strain RCMB008001 was obtained from the Regional Center for Mycology and Biotechnology et al.-Azhar University. The identity of this fungal strain was verified by conducting a pathogenicity test. Three-day-old culture FO was swabbed with a sterilized swab on the surface of PDA plate to test the antifungal activity of the macroalga extract using the agar well diffusion method., The algal extract at (1000 µg/mL) was prepared one hundred micro liters of crude extract was transferred into well and left for 2 h at 4 ℃^13^.

### Gas chromatography-mass spectroscopy (GC–MS) analysis

The metabolites present in the algal ethyl acetate extract was analyzed, counted, and identified using GC–MS, as explained by Sharaf et al.^14^.with minor modifications. Briefly, crude extract was dissolved in spectroscopy-grade methanol. GC–MS analysis was performed on Trace GC1310-ISQ mass spectrometer (Thermo Scientific, Austin, Texas, USA), using a direct capillary column (length 30 m, thickness 0.25 µm, internal diameter 25 mm). The oven temperature was started at 50 ℃ held for 5 min and ramped at 5 ℃ per min up to 230 ℃ and held for 2 min; 1 μL of the sample was injected at 250 ℃ using helium as a carrier gas, split at the ratio of 1:30. The mass spectrometer was run in the electron ionization (EI) mode at 200 ℃ and 70 eV with a scan range of 40 to 1000 m/z. The spectrum of the detected compounds was compared with the spectrum of the known compounds stored in the WILEY 09 (Wiley, New York, NY, USA) and NIST 11 library. The name, molecular weight, and chemical structure of the detected compounds were also determined.

### Experimental location

The study was carried out at the experimental farm at Botany and Microbiology Department, Al-Azhar University, Cairo, Egypt.

### Experimental scheme

Seedlings of the *Solanum lycopersicum L*. variety 023 were obtained from the Giza Agriculture Research Centre in Egypt at the age of four weeks. In a greenhouse, uniform seedlings were transplanted into plastic pots (40X40 cm) with a sand and clay mixture (1:3) weighing a total of 7 kg in the greenhouse. The pots were maintained at a constant 22/18 °C day/night and 70–85% relative humidity. JE was applied 7 days before inoculation with (FO). JE extract was given either through foliage or soil. The details of treatments (4 replicates for each treatment) include set I- healthy control (no fungus); set 2- FOW control; set 3- Healthy + JE (FS); set 4- FOW + JE (FS); set 5- Healthy + JE (SI) set 6*-* FOW + JE (SI); Plants were carefully uprooted 60 days after planting (DAP) and examined for the Disease index calculation and various characteristics mentioned below.

### Vegetative development

Growth indicators such as root length (cm) and Stem height (cm) and number of leaves / plant were estimated post-harvest.

### Disease index (DI)

The disease symptoms were evaluated, and the disease index was calculated using a scale with five classes: 0 (no symptoms), 1 (slight yellowing of lower leaves), 2 (moderate yellowing of the plant), 3 (wilted plant), and 4 (severely stunted and destroyed plants). The disease index (DI) was determined using the formula: DI = [(1 × n1) + (2 × n2) + (3 × n3) + (4 × n4)] × 100 / (4 × Nt), where n1, n2, n3, and n4 represent the number of plants in each respective class, and Nt is the total number of plants evaluated. The severity of the FW disease and the percentage of protection provided by the JE were calculated using the following equation: Protection % = [(A—B) / A] × 100%. In this equation, A represents the Plant Disease Index in infected control tomato plants, and B represents the PDI in JE -treated tomato plants^6^.

### Photosynthetic measurements

Fresh leaf tissue weighing 0.5 g was pulverised in 80% acetone using a pestle and mortar to determine the pigment content. After centrifuging for 5 min at 10,000 rpm (11180 g) (1118RCF) absorbance of filtrate was measured at 470, 652 and 665 nm to estimate chlorophyll^15^, and carotenoid ^16^ by the following equations : mg chlorophyll (a) / g tissue = 11.63(A665) – 2.39(A649), mg chlorophyll (b) / g tissue = 20.11(A649) – 5.18(A665)., Carotenoids = 1000 × O.D_470_- 1.82 C_a_ – 85.02 C_b_/ 198 = mg/g fresh weight.

### Measurement of phenolics

Total phenolics were determined by the process described^17^. The 1.5 mL Folin reagent solution received a 100 µL extract, and the mixture was then incubated at 25 ℃ for 1 min. 1.5 mL of aqueous Na_2_CO_3_ was added and allowed to sit at 25 ℃ for 90 min while kept in the dark. Absorbance was measured at 765 nm.

### Assessment of proline

The Bates et al*.*^18^ method was used to estimate proline. 0.5 g of dry plant material were homogenized in 10 ml of 3% sulfosalicylic acid. The homogenate was filtered, and 2 ml of the filtrate was mixed with 2 ml of acid ninhydrin and 2 ml of glacial acetic acid. The mixture was heated for 1h, then immediately cooled in an ice bath. Four ml of toluene were added to the reaction mixture and mixed vigorously for 0.5 min. The toluene extract was then warmed to room temperature, and the absorbance was measured at 520 nm.

### Test for antioxidant enzymes

The technique developed by Bergmeyer^19^ was used to assess POD activity. Polyphenol oxidase (PPO) activity was recognized by a procedure of the Lavid et al*.*^20^ . Two grams of fresh tomato plant were mixed with 10 ml of pH 6.8 phosphate buffer (0.1 M), homogenized, and preserved as an enzyme source. POD activity was measured by mixing 0.2 ml of the enzyme extract with 5.8 ml of 50 mM phosphate buffer (pH 7) and 2 ml of 20 mM H_2_O_2_. After adding 2 ml of 20 mM pyrogallol, the increase in absorbance was monitored at 470 nm. PPO activity was determined by mixing 125 µmol of phosphate buffer (pH 6.8), 100 µmol of pyrogallol, and 2 ml of the enzyme extract. After a 5 min incubation at 25 ℃, 1 ml of 5% H_2_SO_4_ was added. The resulting color was measured at 430 nm.

### Isozymes electrophoresis

Electrophoresis of isozymes was conducted using native polyacrylamide gel to observe modifications in response to treatments. The peroxidase (POD) and polyphenol oxidase (PPO) isozymes was evaluated using^4^.

### Statistical analysis

The results presented are the means ± standard error of three replicates (n = 3). These results were statistically confirmed by analysis of variance (ANOVA LSD test at p < 0.05 was employed by CoStat to demonstrate the substantial variances between treatments. The findings (n = 3) were presented as an average for typical errors.

### Plant collection

The plant collection and use were in accordance with all the relevant guidelines.

## Results

### Algal identification

**Figure 1:**
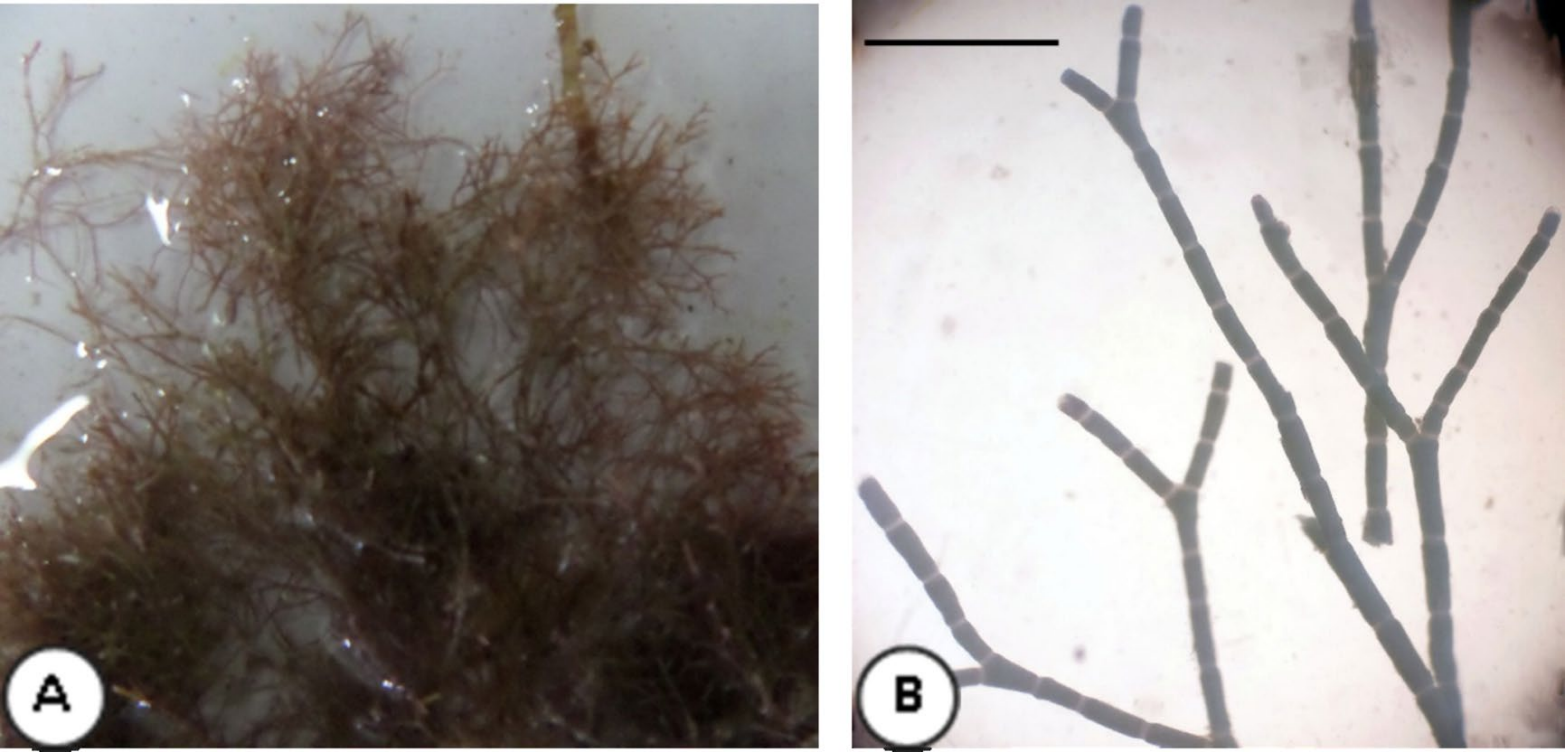
(**A–B**). Morphological characteristics of the red seaweed *Jania* sp. [Scale bar = 1 mm].

The red macroalga (Figs. 1 A-B) showing thallus and dichotomously branched, with segments and identified as *Jania* sp.

### GC–MS analysis of the JE

**Figure 2:**
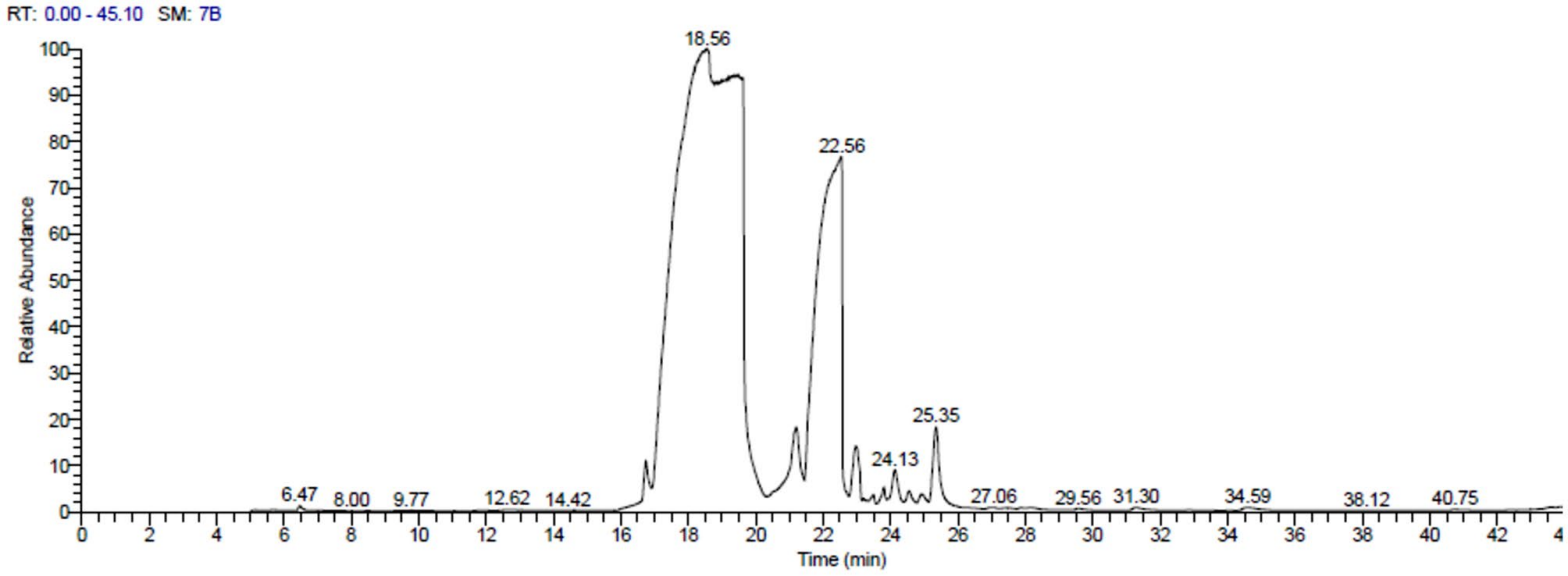
GC–MS chromatogram of JE.

The GC–MS analysis provides a typical spectral output for each compound found in the analysed samples. The GC–MS analysis of JE revealed that it contained approximately 35 compounds, with more than 70% are minor compounds. The highest peak area at 18.56 min with the presence of Eugenol (43.19%), followed by Phenol,2-methoxy-4-(2-propenyl)-,acetate (35.89%), Caryophyllene oxide (5.77%), and Isoespintanol (3.69%), as shown in Table 1 and Fig. 2.

**Table 1:** Major compound detected in GC Mass of JE.

No	Compound name	RT (min)	Peak area %	activity	Ref
1	Copaene	16.73	1.48	antioxidant, antifungal	^21^
2	Phenol, 2-methoxy-4-(2-propenyl)	17.57	3.23	Plant growth promoting	^22^
3	Eugenol	18.56	43.19	Atifungal, Antibacterial and antiviral	^23^
4	Cadinene	21.20	2.98	antitumor activity	^24^
5	Phenol, 2-methoxy-4-(2-propenyl)-,acetate	22.56	35.89	Antimicrobial	^25^
6	Caryophyllene oxide	24.13	5.77	Anticancer	^26^
7	Isoespintanol	25.35	3.69	antibacterial and antibiofilm	^27^

### In-vitro antifungal activity of JE

**Figure 3:**
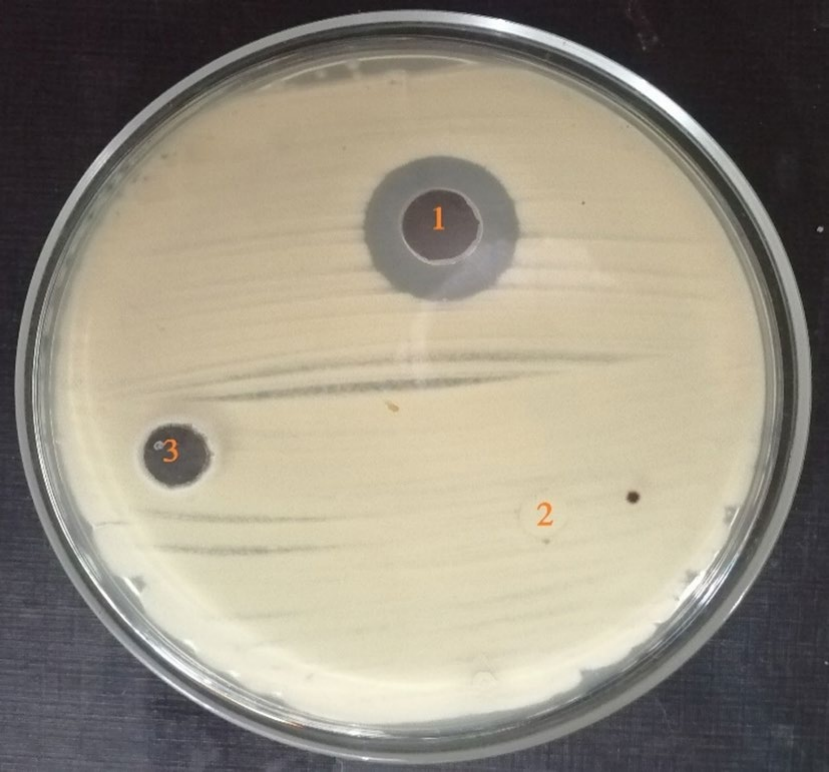
Antifungal activity of JE on *F. oxysporum* ;(1) JE (2) Antifungal disc control fluconazole,(3) sterile distilled water in well.

The results observed in Fig. 3 indicate that JE has antifungal activity against FO, with an inhibition zone diameter of 20 mm. On the other hand, negative control had no effect on FO, which proves the exclusive activity of JE. Antifungal control (fluconazole) did not show activity against FO.

### Percent disease incidence (PDI) and Percent protection (P%)

**Figure 4:**
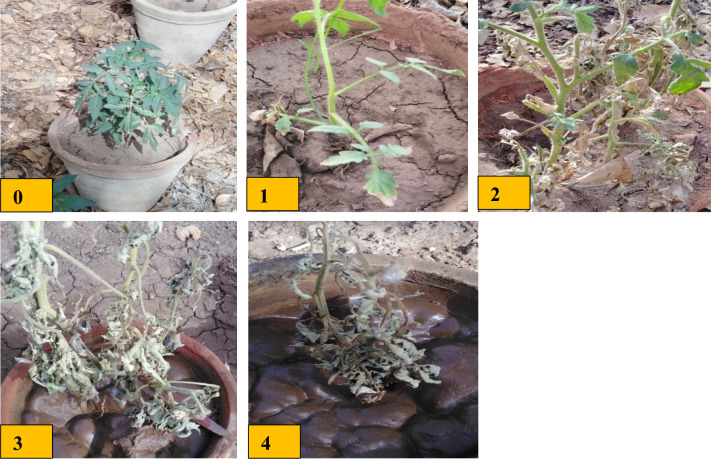
Five classes of *Fusarium* wilt disease: (**0**) (no symptoms), (**1**) (slight yellowing of lower leaves), (**2**) (moderate yellowing of the plant), (**3**) (wilted plant), and (**4**) (severely stunted and destroyed plants).

Table 2 and Fig. 4 reveal that the application of JE, either through foliar or soil, significantly reduced FOW PDI compared with infected control. Conversely, According to the results, infected control plants had a 91.6% PDI. foliar shoot by JE was the most effective treatment, reducing PDI by 20.83% and caused high protection by 77.25%. In the following, the soil irrigation by *Jania* algal extract reduced PDI by 33.33% and caused high protection by 63.61%.

**Table 2:** Effect of JE on DI.

Treatment	Disease symptoms classes					DI (disease index) (%)	Protection (%)
	0	1	2	3	4		
Control healthy	6	0	0	0	0	0	–
Control Infected	0	0	0	2	4	91.6	0
Infected + JE Foliar	2	3	1	0	0	20.83	77.25
Infected + JE drainage	2	2	0	2	0	33.33	63.61

### Growth biomarkers

Table 3 shows that JE treatments significantly affected various growth indices, including shoot length (SL), root length (RL), and leaf number (LN). Control tomato plants with FOW showed a marked decrease in SL, lower RL, and fewer leaves. Moreover, these plants lost more LN (60.1%), SL (26.8%), and RL (51.7% compared to healthy control plants). Conversely, healthy plants treated with JE through different methods, like soil and foliar spray, exhibited potential improvement in a variety of growth indices such as SL, RL, and LN. On the other hand, diseased plants treated with JE through various modes, such as foliar spray and soil application, shown encouraging recovery. In comparison to two modes, it was discovered that applying JE through foliar spray and soil irrigation to infected plants successfully restored the losses in SL (35 and 22%), RL (49.1 and 26.83%), and LN (31.6 and 12.5%).

**Table 3:** Morphological markers of a JE -treated tomato plant.

Treatment	SL (cm)	RL (cm)	LN/plant
Control healthy	27.33 ± 2.5^bc^	9.66 ± 0.57^bc^	52.66 ± 8.3^b^
Control Infected	20 ± 1.52^c^	4.66 ± 0.55^d^	21 ± 3.6^c^
Healthy + JE Foliar	55.66 ± 5.13^a^	15.66 ± 3.2^a^	64.33 ± 5.8^a^
Infected + JE Foliar	27 ± 1^bc^	9.16 ± 0.53^bc^	30.66 ± 2.08^c^
Healthy + JE drainage	33 ± 1.03^b^	12 ± 1^b^	60.5 ± 3.53^ab^
Infected + JE drainage	25.66 ± 7.23^bc^	6.33 ± 1.57^cd^	24 ± 1^c^
LSD at 0.05	6.86	2.781	10.662

Letters a: d (significant letters).

*SL Shoot length, *RL* Root length, *LN* Leaves numbers.

### Photosynthetic pigments

Plant FOW resulted in a decline in components Chl. a and Chl. b. Comparing the samples to the healthy control plants, the carotene content did not significantly increase. As opposed to healthy control plants, a good recovery response was seen when healthy plants were treated with JE using various modalities. While JE through soil application recovered the loss of carotene content, JE through foliar spray was successful in recovering the loss of Chl. a and Chl. B (Table 4).

**Table 4:** Photosynthetic pigments of tomato plant treated with JE.

Treatment	Chlorophyll a ( mg/ g fresh weight )	Chlorophyll b ( mg / g fresh weight)	Carotene ( mg/ g fresh weight )
Control healthy	9.54 ± 0.21^e^	3.430.38 ± ^d^	1.13 ± 0.39^b^
Control Infected	7.15 ± 0.1 f.	5.86 ± 1.45^c^	1.47 ± 0.31^b^
Healthy + JE Foliar	21.21 ± 0.2^a^	21 ± 0.80^a^	5.2 ± 2.49^a^
Infected + JE Foliar	12.79 ± 1.02^c^	8.26 ± 0.59^b^	4.21 ± 0.94^a^
Healthy + JE drainage	18.450.19 ± ^b^	9.58 ± 0.33^b^	4.9 ± 0.16^a^
Infected + JE drainage	11.050.05 ± ^d^	6.84 ± 0.36^c^	2.71 ± 0.15^ab^
LSD at 0.05	0.79	1.35	1.98

*Data represent mean ± SD, n = 3. (a: f revealed to significance letters).

### Total phenolics and proline

**Figure 5:**
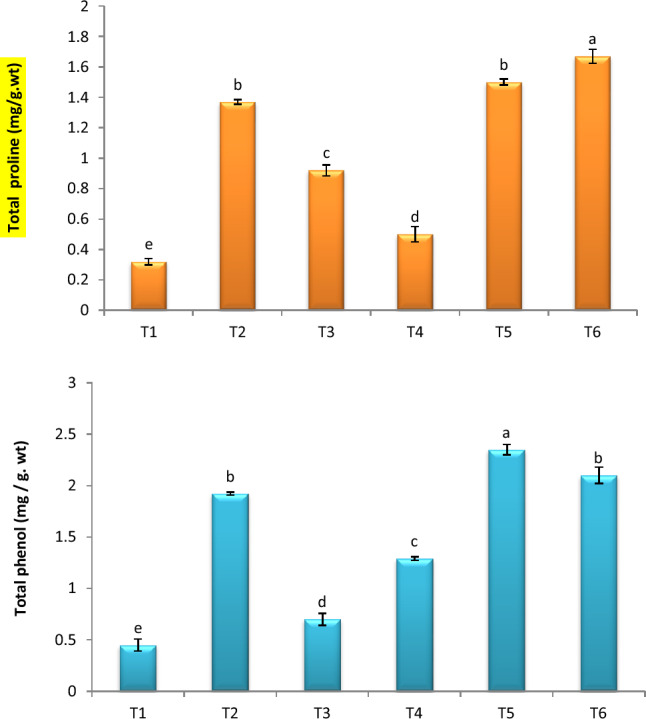
Effect of JE on total phenol and proline. TI- healthy control (no fungus) ; T2- FOW infected control; T3- Healthy + JE (FS); T4- FOW + JE (FS); T5- Healthy + JE (SI) , and T6*-* FOW + JE (SI).

The total phenol and proline concentrations in the tomato plants were significantly higher with FOW compared to control plants (Fig. 5). The untreated healthy plants showed minimum values for total phenol content in comparison to FOW plants. Total phenol content, however, increased in JE -treated plants, reaching highest levels in infected plants treated with JE through foliar application, followed by JE through soil (Fig. 5). Infected plants treated with JE through soil treatment had the highest proline concentration, followed by plants treated with JE by foliar application (Fig. 5).

### Activity of polyphenol oxidase (PPO) and peroxidase (POD)

**Figure 6:**
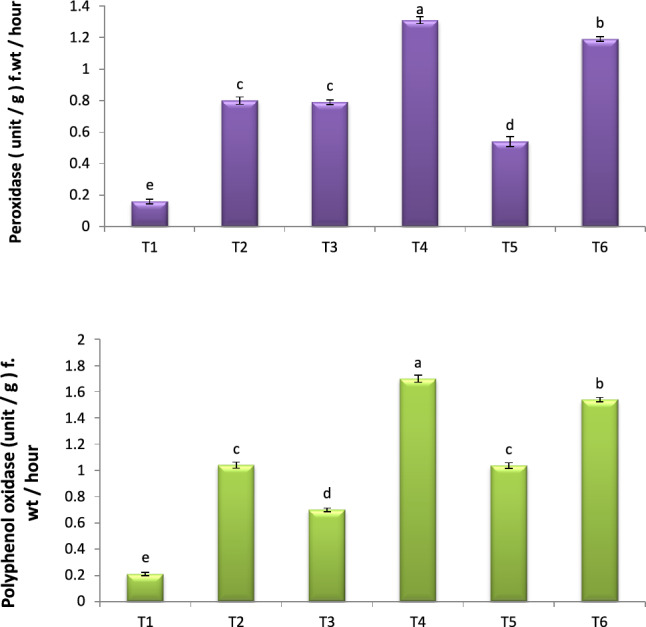
Effect of JE on antioxidant enzymes. Effect of JE on total phenol and proline. TI- healthy control (no fungus) ; T2- FOW infected control; T3- Healthy + JE (FS); T4- FOW + JE (FS); T5- Healthy + JE (SI) , and T6*-* FOW + JE (SI).

In control plants, POD and PPO activity was barely detectable. Figure 6 clearly shows that POD and PPO activity increased dramatically in response to FO and/or JE. When JE was administered to FOW plants, the activity of POD and PPO also rose dramatically. The highest concentrations of these enzymes were discovered in infected plants that were treated with JE through foliar application initially, followed by JE through soil.

### Antioxidant isozymes ( POD & PPO)

**Figure 7:**
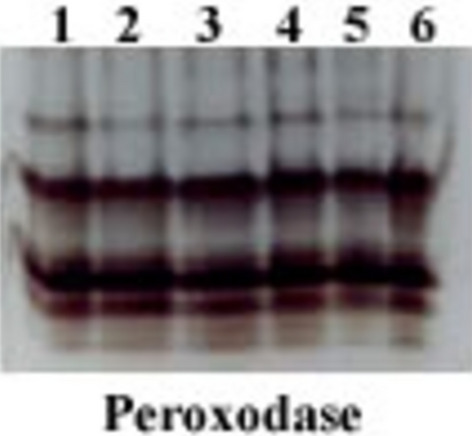
Effect of FO and/or JE and their interactions on peroxidase isozyme I- healthy control (no fungus); 2- FOW infected control; 3- Healthy + JE (FS); 4- FOW + JE (FS); 5- Healthy + JE (SI) , and 6*-* FOW + JE (SI).

Native PAGE in (Fig. 7 ) and Table 5 showed Six POD isozymes at Rf values 0.470, 0.634, 0.845, 0.903, 0.938 and 0.968. POD activity was much higher in infected plants compared to healthy control plants. FOW Infected plants exhibited 6 bands, including three week bands at Rf values 0.903, 0.938 and 0.968, A moderate band at Rf 0.634 and highly dense band at Rf 0.845.

**Table 5:** Isomers of peroxidase enzymes (+ / −) and their Retention factor (Rf) in response to *Fusarium*, foliar application of *Jania* sp. and their interactions on tomato plants.

RF	Control healthy	Control infected	Healthy + JE Foliar	Infected + JE Foliar	Healthy + JE soil	Infected + JE soil
0.470	+	+	+	+	+	+
0.634	+	+ +	+ +	+ +	+ +	+ +
0.845	+ +	+ + +	+ + +	+ + +	+ + +	+ + +
0.903	+	+	+	+ +		+ +
0.938	+	+	+	+	+	+

The same six bands were recorded at the same Rf in infected plants treated with JE through different modes, such as soil and foliar spray, with two of them being moderated bands at Rf (0.634 and 0.903), while the other three bands were faint at Rf (0.470, 0.938, and 0.968), and one highly dense band at Rf values 0.845. Untreated control plants produced the fewest POD bands, 5, of which four were faint bands at Rf (0.634 and 0.903), three were faint at Rf (0.470, 0.634, 0.903, and 0.938), and one was moderately dense at Rf 0.845.

**Figure 8:**
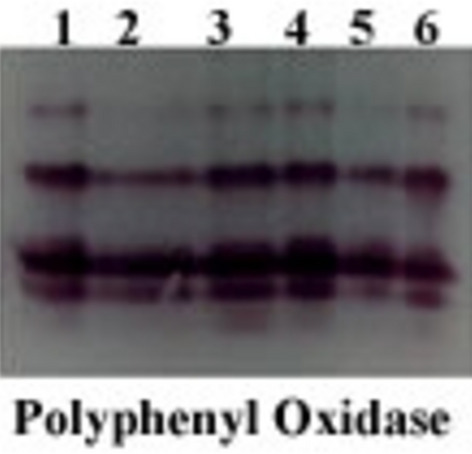
Effect of FO and/or JE and their interactions on PPO isozyme. I- healthy control (no fungus); 2- FOW infected control; 3- Healthy + JE (FS); 4- FOW + JE (FS); 5- Healthy + JE (SI) , and 6*-* FOW + JE (SI).

The PPO isozyme of tomato plant leaves at Rf contained four PPO isozymes (0.274, 0.492, 0.782, and 0.883). JE was applied to WOF plants for substantially overexpressed PPO, which recorded four bands, two of which were moderate bands at Rf (0.492 and 0.883), as well as two faint bands at Rf (0.274) and one extremely dense band at Rf (0.782). Healthy plants treated with JE through soil showed less expressed PPO that recorded 3 bands all of them was faint bands at Rf (0.492 0.782 and 0.883) as shown in Fig. 8 and Table 6.

**Table 6:** Isomers of polyphenol oxidase enzymes (+ / −) and their Retention factor (Rf) in response to *Fusarium*, foliar application of *Jania* sp.and their interactions on tomato plants.

RF	Control healthy	Control infected	Healthy + JE Foliar	Infected + JE Foliar	Healthy + JE soil	Infected + JE soil
0.274	+	−	+	+	−	+
0.492	+	+	+ +	+ +	+	+ +
0.782	+ +	+ + +	+ + +	+ + +	+	+ + +
0.883	+ +	+ +	+ +	+ +	+	+

## Discussion

Numerous Notable bioactive ingredients have been discovered in red algae, including polysaccharides , lipids, glycosides, steroids, flavonoids, polyphenols, tannins, triterpenoids, alkaloids, and antheraquinones. They have a special ability to successfully halt the growth of many pathogenic microorganisms, particularly fungi^28^.

Protein, lipids, and carbohydrates are just a few of the main dietary components that constitute *Jania*. In aquaculture, the nutritional value of dietary seaweed supplements is determined by growth performance, feed consumption, and survival rate. Researchers have discovered that seaweed can be used as a biocontrol agent for diseases in both humans and plants due to its a strong vitamin and mineral profile, is particularly high in ascorbic acid content, and a number of active substances^29^.

GC–MS analysis of JE revealed that Eugenol, phenol,2-methoxy -4- (2-propenyl) -,acetate, caryophyllene oxide, isoespintanol are compounds recognized for having antifungal activity. The usefulness of a novel supplement, JE, in minimizing the detrimental effects of FOW tomato plants was examined in the current study. Wilt disease is regarded as one of the most detrimental biotic factors affecting development and productivity^30^. Effective preventative measures have been created to lessen the harmful effects by eliminating the pathogen or enhancing plant resistance^31^. The addition of novel mitigating chemicals can enhance the growth and productivity of plant under biotic stress^32^. FO attacks a plant’s internal vascular system . Instead of altering the entire soil micro flora, it is preferable to use biotic inducers as a safety measure for people and the environment to safeguard the spot where this fungus enters the plant^33^.

When plants were treated with JE, the proportion of disease severity decreased, and their resistance to FOW and development of systemic resistance in plants increased. The application of JE under FOW significantly dramatically increased SL, RL, and NL. In the current study, FO dramatically decreased plant growth. The cumulative effects of FO on critical metabolic and enzyme functioning lead to a reduction in morphological characteristics and stresses hampering the cell cycle progression^34^.

Using biotic inducers for improving growth has been proposed as promising management technique for crop improvement. However, it is quite uncommon to find reports on JE advantageous effects on plants. The use of JE in the suggested method significantly increased the levels of chlorophyll and enhanced plant development. It was discovered in this study that using JE helped to advance the process of creating chlorophyll pigments. By mediating ROS scavenging and assisting in redox maintenance, the increased synthesis of carotenoids caused by the administration of JE possibly aided in photosynthetic defence. The use of bio stimulators increased the synthesis of carotenoids and chlorophyll^35^. It was shown that FOW dramatically enhanced POD and PPO activity^36^. Others have found similar further enhanced antioxidant activity^37^. The application of JE significantly enhanced POD and PPO activity. Previously, it has been observed that biotic inducers improve the performance of plants such as tomatoes as antioxidants^35^. The protection of photosynthesis, enzyme activity, and membrane integrity is facilitated by up regulated antioxidant enzyme activity, which maintains plant performance^38^. Although foliar applied JE was more helpful, both types of JE treatment had substantial effects. H_2_O_2_ is eliminated by peroxidases at membranes^39^. Application of an amino acid-rich weed extract significantly enhanced the antioxidant performance of beans, leading to increased phenol, flavonoid accumulation^40,41^. Through improved stabilisation of cellular structures and enzyme activity in important metabolic pathways, JE-mediated rise in the proline content might have aided in cellular growth and maintenance^42^. The accumulated proline could be distributed throughout the cytoplasm to balance osmotic pressure from the vacuoles and protect bio macromolecules^43,44^ (Supplementary information).

## Conclusion

This study establishes that *Fusarium oxysporum* wilt (FOW) causes oxidative damage, leading to decreased physiological performance and growth in tomato plants. However, immune responses improved and antioxidant enzymes were up regulated when Jania sp. ethyl acetate extract (JE) was applied to the plants affected by FOW through foliage or soil. Additionally, plants exposed to JE exhibited an increase in osmoprotectants (proline) and phenolic compounds, both of which act as scavengers to eliminate excess reactive oxygen species during FOW infection. The study supports the beneficial application of JE in protecting tomato plants against FOW, although further research is necessary to elucidate the specific mechanisms involved.

### Supplementary Information

Supplementary Information 1.

Supplementary Information 2.

Supplementary Information 3.

Supplementary Information 4.

Supplementary Information 5.

Supplementary Figures.

Supplementary Information 6.

Supplementary Information 7.

## Data Availability

The datasets used and/or analyzed during the current study available from the corresponding author on reasonable request**.**

## Abbreviations

JE: *Jania* Sp. ethyl acetate extract
FOW: *Fusarium* *oxysporum* Wilt
FO: *Fusarium* *oxysporum*
SI: Soil irrigation
FS: Foliar shoots
DIP: Disease index present
GC–MS: Gas chromatography-mass spectroscopy
PPO: Polyphenol oxidase
POD: Peroxidase
