# Author Correction: Enhancing tomato plant immune responses to Fusarium wilt disease by red seaweed Jania sp.

**DOI:** 10.1038/s41598-024-71361-y

**Published:** 2024-08-30

**Authors:** Amer M. Abdelaziz, Ahmed A. Elrefaey, Mohamed H. Sharaf, Rahmah N. Al-Qthanin, Mohamed S. Attia

**Affiliations:** 1 Botany and Microbiology Department, Faculty of Science, Al-Azhar University, Cairo, 11884 Egypt; 2 Biology Department, College of Sciences, King Khalid University, Abha, Saudi Arabia; 3 Prince Sultan Bin-Abdul-Aziz Center for Environmental Researches and Natural Resources Sustainability, King Khalid University, P.O. Box 960, 61421 Abha, Saudi Arabia

Correction to: *Scientific Reports* 10.1038/s41598-024-67233-0, published online 05 August 2024

The original version of this Article contained an error in Conclusion.

“However, immune responses improved and antioxidant enzymes were up regulated when Jasmonic Acid Ester (JE) was applied to the plants affected by FOW through foliage or soil.”

now reads:

“However, immune responses improved and antioxidant enzymes were up regulated when Jania sp. ethyl acetate extract (JE) was applied to the plants affected by FOW through foliage or soil.”

The original Article has been corrected.

