# Supplementary figures and images for: Enhancing tomato plant immune responses to Fusarium wilt disease by red seaweed Jania sp
Source: Sci Rep. 2024 Aug 5;14:18052. doi: 10.1038/s41598-024-67233-0 (PMC11300823; doi:10.1038/s41598-024-67233-0)

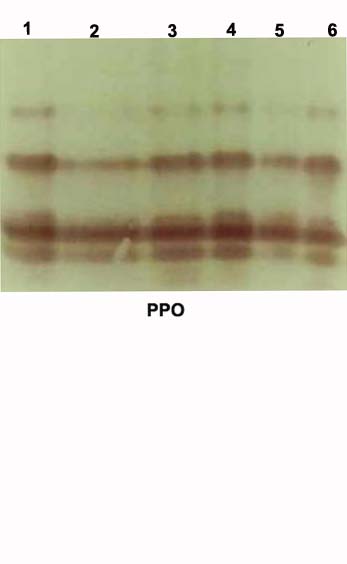

Supplement: Supplementary file 1 — Supplementary Information 1. [file 41598_2024_67233_MOESM1_ESM.jpg]

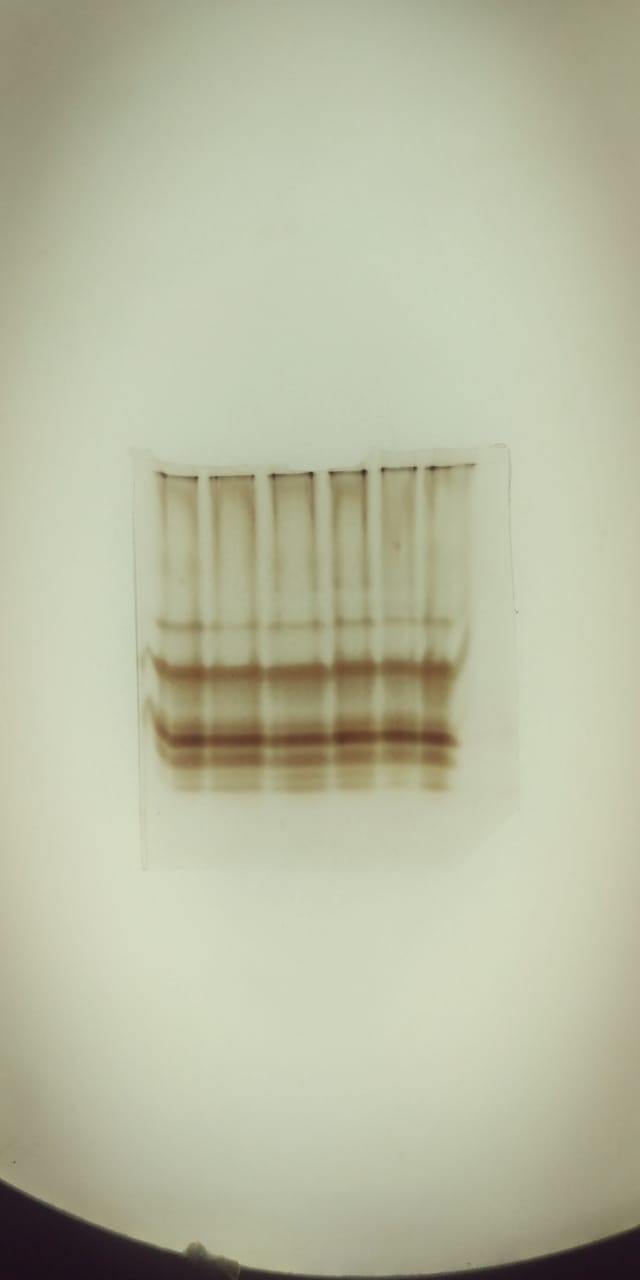

Supplement: Supplementary file 3 — Supplementary Information 3. [file 41598_2024_67233_MOESM3_ESM.jpeg]

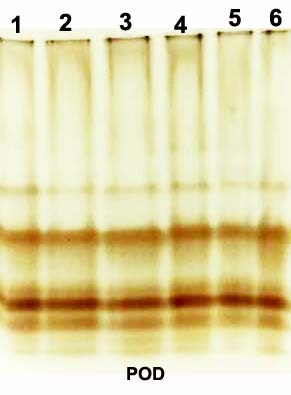

Supplement: Supplementary file 4 — Supplementary Information 4. [file 41598_2024_67233_MOESM4_ESM.jpg]

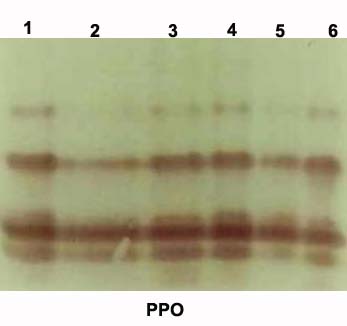

Supplement: Supplementary file 7 — Supplementary Information 6. [file 41598_2024_67233_MOESM7_ESM.jpg]

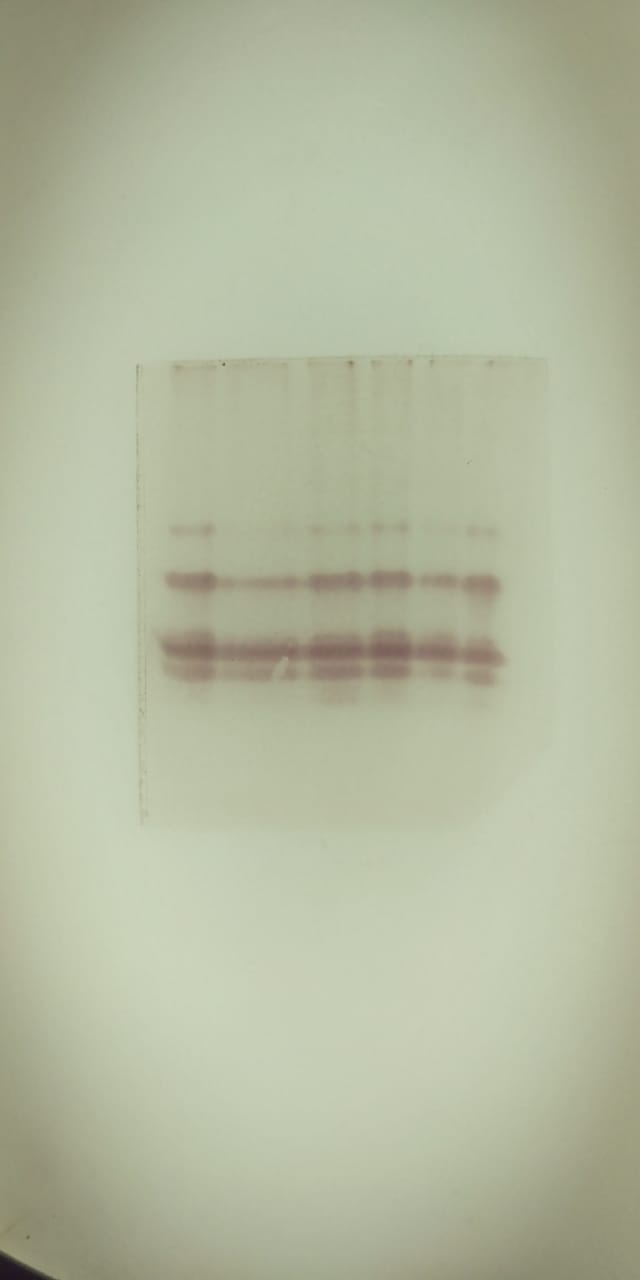

Supplement: Supplementary file 8 — Supplementary Information 7. [file 41598_2024_67233_MOESM8_ESM.jpeg]
