# Supplementary material for: Enhancing tomato plant immune responses to Fusarium wilt disease by red seaweed Jania sp
Source: Sci Rep. 2024 Aug 5;14:18052. doi: 10.1038/s41598-024-67233-0 (PMC11300823; doi:10.1038/s41598-024-67233-0)
Supplement: Supplementary file 6 — Supplementary Figures. [file 41598_2024_67233_MOESM6_ESM.docx]

**Enhancing tomato plant immune responses to *Fusarium* wilt disease by red *seaweed Jania*** sp.


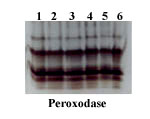


**Figure 7. Effect of FO and/or JE and their interactions on peroxidase isozyme I- healthy control (no fungus) ; 2- FOW infected control; 3- Healthy + JE (FS); 4- FOW + JE (FS); 5- Healthy + JE (SI) , and 6*-* FOW + JE (SI).**


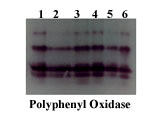


**Figure 8**. **Effect of FO and/or JE and their interactions on polyphenol oxidase isozyme. I- healthy control (no fungus) ; 2- FOW infected control; 3- Healthy + JE (FS); 4- FOW + JE (FS); 5- Healthy + JE (SI) , and 6*-* FOW + JE (SI).**
